# Coronavirus disease 2019 (COVID-19) universal admission screening in patients and companions in Taiwan from May 2021 to June 2022: A nationwide multicenter study

**DOI:** 10.1017/ice.2023.144

**Published:** 2024-01

**Authors:** Hao-Hsin Wu, Chiu-Hsia Su, Li-Jung Chien, Shu-Hui Tseng, Shan-Chwen Chang

**Affiliations:** 1 Division of Infection Control and Biosafety, Taiwan Centers for Disease Control, Taipei, Taiwan; 2 Department of Internal Medicine, National Taiwan University Hospital, Taipei, Taiwan; 3 College of Medicine, National Taiwan University, Taipei, Taiwan

## Abstract

**Objective:**

Universal admission screening and follow-up symptom-based testing for severe acute respiratory syndrome coronavirus 2 (SARS-CoV-2) may play critical roles in controlling nosocomial transmission. We describe the performance of test strategies for inpatients and their companions during various disease incidences in Taiwan.

**Design:**

Retrospective population-based cohort study.

**Setting:**

The study was conducted across 476 hospitals in Taiwan.

**Methods:**

The data for both testing strategies by reverse transcription-polymerase chain reaction for SARS-CoV-2 in newly admitted patients and their companions during May 2021—June 2022 were extracted and analyzed.

**Results:**

The positivity rate of universal admission screening was 0.76% (14,640 of 1,928,676) for patients and 0.37% (5,372 of 1,438,944) for companions. The weekly community incidences of period 1 (May 2021–June 2021), period 2 (July 2021–March 2022), and period 3 (April 2022–June 2022) were 6.57, 0.27, and 1,261, respectively, per 100,000 population. The positivity rates of universal admission screening for patients and companions (4.39% and 2.18%) in period 3 were higher than those in periods 1 (0.29% and 0.04%) and 2 (0.03% and 0.003%) (all *P* < .01). Among the 22,201 confirmed cases, 9.86% were identified by symptom-based testing. The costs and potential savings of universal admission screening for patients and companions achieved a breakeven point when the test strategy was implemented in a period with weekly community incidences of 27 and 358 per 100,000 population, respectively.

**Conclusions:**

Universal admission screening and follow-up symptom-based testing is important for reducing nosocomial transmission. Implementing universal admission screening at an appropriate time would balance the benefits with costs and potential unintended harms.

Healthcare-associated coronavirus disease 2019 (COVID-19), caused by severe acute respiratory syndrome coronavirus-2 (SARS-CoV-2), is a significant threat to vulnerable hospitalized patients with severe outcomes and have imposed critical pressure on hospital operations.^1–6^ One of the leading causes of widespread transmission of SARS-CoV-2 is the infectivity of asymptomatic and presymptomatic infections, which might account for 30%–85% of SARS-CoV-2 infections, varying with the epidemiological setting and case mix.^7,8^ Unidentified infections present a unique challenge to infection control in healthcare settings, and their early identification may help contain the in-hospital transmission of SARS-CoV-2 with concomitant infection prevention and control (IPC) measures, including isolation of cases, quarantine of contacts, and the appropriate use of personal protective equipment (PPE).^3,9,10^ Therefore, universal admission screening for patients upon admission to identify asymptomatic or presymptomatic individuals has been adopted as an IPC measure, depending on the disease prevalence and specific targeted populations.^7,11,12^

Nevertheless, the universal admission screening strategy might also pose challenges for healthcare facilities, such as the high cost of tests, long test turnaround time, additional requirements for the workforce, and PPE required to perform the tests.^3,5,13–15^ Moreover, the potential benefits of universal admission screening should be balanced with the unintended harmful consequences to patients, such as the risk of delaying appropriate treatments or incurring unnecessary isolation due to false-positive results or lingering positivity from a past infection.^7,11,15–17^ Hence, whether universal admission screening is justified remains controversial in areas with a low prevalence of COVID-19, where the positive detection rate is relatively low, and the cost-effectiveness is unclear.^3,5,10,15,16^

Since the global COVID-19 pandemic, Taiwan has experienced 2 waves of widespread COVID-19 circulation in the community as of June 2022.^18^ The first wave peaked between May and July 2021, followed by low community transmission. The second wave occurred from April to June 2022. To respond to the COVID-19 epidemic, the Central Epidemic Command Centre (CECC) implemented reverse transcription polymerase chain reaction (RT-PCR)–based universal admission screening for SARS-CoV-2 in newly admitted patients and their companions, in addition to symptom-based testing as a multifaceted mitigation measure.^1,19^ Hospitalized patients and their companions were tested for SARS-CoV-2 within 48 hours prior to or on the day of admission. We compared the effectiveness of universal admission screening and follow-up symptom-based testing for newly admitted patients and their companions and to estimate the costs and potential savings provided by universal admission screening during different disease incidences in Taiwan.

## Methods

### Study design and setting

This nationwide population-based observational study was conducted retrospectively. COVID-19 has been classified as a notifiable communicable disease in Taiwan since January 15, 2020.^1^ According to clinician assessments, all patients with COVID-19–related symptoms could be tested for SARS-CoV-2 by RT-PCR at public expense. On May 17, 2021, the CECC provided government-funded, RT-PCR–based, universal admission screening for SARS-CoV-2, with different billing codes from symptom-based testing, in newly admitted asymptomatic patients and their companions within 48 hours prior to or on the admission day. We analyzed the results of universal admission screening and follow-up symptom-based testing for SARS-CoV-2 in hospitalized patients and their companions and characterized cases with positive results of either of the tests at 476 hospitals in Taiwan from May 2021 to June 2022.

For follow-up symptom-based testing, only tests performed within 7 days after the date of universal admission screening that yielded negative results were included in this study. The tests for previously known COVID-19 cases were excluded.

### Assumptions and parameters for evaluation of investment costs and financial benefits of universal admission screening

We assumed that unidentified infectious COVID-19 cases, either patients or companions, once entering hospitals for admission or accompanying, would initiate healthcare-associated COVID-19 transmissions, which the early identification of index cases with universal admission screening could avert. Thus, the financial benefits attributable to universal admission screening are considered the potential savings related to healthcare-associated transmissions averted, comprising medical expenses of caring for subsequently transmitted COVID-19 cases and costs of immediate RT-PCR-based screening for close contacts.

According to the database of our previous investigation of healthcare-associated COVID-19 outbreaks in Taiwan,^1^ an unidentified inpatient COVID-19 case would incur a healthcare-associated COVID-19 outbreak with 7.5 subsequent cases (including 3.7 inpatients and 3.8 outpatients) and 143 close contacts on average. In comparison, a companion case would contribute to 5 subsequent cases (including 1.7 inpatients and 3.3 outpatients) and 94 close contacts on average. According to the National Health Insurance (NHI) declaration database, the average medical expenses for caring for COVID-19 cases were estimated at $1,808 per inpatient case and $100 per outpatient case. The cost of the government-funded SARS-CoV-2 RT-PCR test paid to hospitals by the CECC was $100 per test (covering material and staff costs).

### Definitions

We divided the study period into 3 periods: period 1, from May 2021 to June 2021, when the average weekly number of new domestic cases was 6.57 per 100,000 population; period 2, from July 2021 to March 2022, when the average weekly number of new domestic cases was 0.27 per 100,000 population; and period 3, from April 2022 to June 2022, when the average number of new weekly domestic cases was 1,261 per 100,000 population. The main SARS-CoV-2 variant that circulated during periods 1 and 2 was the α (alpha) variant; however, during period 3, the omicron variant dominated.^20^ A confirmed case of COVID-19 was defined as an individual with a positive RT-PCR test result for SARS-CoV-2. We categorized the tests and confirmed COVID-19 cases into inpatient and companion groups. The 30-day all-cause mortality was defined as death occurring within 30 days of the date of the first positive RT-PCR test result. Individuals were deemed fully vaccinated 14 days after completing the full primary series of COVID-19 vaccines. The number needed to test (NNT) was defined as the number of persons who required testing to identify 1 additional COVID-19 case. The breakeven point of universal admission screening was defined as a condition in which the investment costs of universal admission screening equaled the cost savings of averted healthcare-associated outbreaks.

### Data collection

In this study, information on the date, result, indication of tests, and personal identity was obtained from the NHI claims data. The characteristics, vaccination status, death, and SARS-CoV-2 test results of the confirmed COVID-19 cases were obtained from the National Infectious Disease Reporting System. The number of close contacts of confirmed cases in healthcare-associated COVID-19 outbreaks was obtained from the Infectious Disease Contact Tracing Platform and Management System.^21^

### Statistical analysis

Categorical variables were compared using the χ^2^ test, and continuous variables were compared using parametric or nonparametric tests. We compared the characteristics of the confirmed cases identified by universal admission screening with those identified by symptom-based testing using multivariate logistic regression. We used logarithm transformation to address the skewed data of weekly community incidence rates and screening positivity rates. The correlation between weekly community incidence rates and screening positivity rates was estimated using simple linear regression. Statistical significance was set at *P* < .05. Statistical analyses were performed using R version 4.1.2 software (R Core Team, R Foundation for Statistical Computing, Vienna, Austria).

### Ethics statement

This study was conducted according to the Declaration of Helsinki protocol and approved (approval no. TwCDCIRB109206; approval date, September 6, 2021) a priori by the institutional review board of the Taiwan Centers for Disease Control. The institutional review board approved the exemption of informed consent because of the retrospective nature of the study.

## Results

During the study period, 3,405,365 government-funded SARS-CoV-2 RT-PCR tests were performed in 476 hospitals, including 3,367,620 (98.89%) admission screening tests performed with a positivity rate of 0.59% (95% confidence interval [CI], 0.59–0.60) and 37,745 (1.11%) symptom-based tests performed with a positivity rate of 5.80% (95% CI, 5.56–6.04). The group-specific and period-specific test numbers, positivity rates, and NNT for universal admission screening and symptom-based testing are presented in Table 1. The positivity rate ratios between symptom-based testing and universal admission screening for patients and companions were 5.32 (95% CI, 4.68–6.05) and 40.31 (95% CI, 34.66–46.88), respectively.

**Table 1.** Universal Admission Screening and Follow-Up Symptom-Based Testing for SARS-CoV-2 in Patients and Companions in Hospitals in Taiwan, May 2021–June 2022

Period	Patients		Companions	
	Universal Admission Screening	Follow-Up Symptom-Based Testing^a^	Universal Admission Screening	Follow-Up Symptom-Based Testing^a^
Period 1 (May–June 2021)^b^				
No. of tests	60,076	2,193	36,846	149
Positive tests, no.	172	7	14	3
Positivity rate, %^c^	0.29	0.32	0.04	2.01
NNT^d^	349	313	2,632	50
Period 2 (July 2021–March 2022)^b^				
No. of tests	1,548,504	19,551	1,157,306	2,806
Positive tests, no.	422	17	30	4
Positivity rate, %^c,^^e^	0.03	0.09	0.00	0.14
NNT^d^	3,669	1,150	38,577	702
Period 3 (April 2022–June2022)^b^				
No. of tests	320,096	9,967	244,792	3,079
Positive tests, no.	14,046	1,257	5,328	901
Positivity rate, %^c,^^e^	4.39	12.61	2.18	29.26
NNT^d^	23	8	46	3
Total (May 2021–June 2022)				
No. of tests	1,928,676	31,711	1,438,944	6,034
Positive tests, no.	14,640	1,281	5,372	908
Positivity rate, %^f^	0.76	4.04	0.37	15.05
NNT	132	25	268	7

Note. COVID-19, coronavirus disease 2019; NNT, number needed to test to identify 1 positive patient; SARS-CoV-2, severe acute respiratory syndrome coronavirus 2.

a Follow-up symptom-based testing was defined as the reverse-transcription polymerase chain reaction tests performed in individuals with suspected symptoms of COVID-19 within 7 days of negative results of universal admission screening.

b Period 1 (May 2021–June 2021), when the number of new average weekly domestic cases was 6.57 per 100,000 population); period 2 (July 2021–March 2022), when the number of new average weekly domestic cases was 0.27 per 100,000 population; and period 3 (April 2022 to June 2022), when the number of new average weekly domestic cases was 1,261 per 100,000 population.

c The positivity rates of universal admission screening and follow-up symptom-based testing for patients and companions in period 3 were significantly higher than those of their counterparts in periods 1 and 2 (all *P* <.01).

d The number needed to test (NNT) was defined as the number of persons who required testing to identify 1 additional case of COVID-19.

e In periods 2 and 3, the positivity rates of follow-up symptom-based testing for patients and companions were significantly higher than those of universal admission screening for their counterparts (all *P* < .01).

f The positivity rates of universal admission screening for patients were significantly higher than that for companions; while the positivity rates of follow-up symptom-based testing for patients were significantly lower than that for companions.

Among 22,201 COVID-19–confirmed cases (median age, 53 years; interquartile range, 37–66 years) identified in this study, 20,012 (90.14%) were detected by universal admission screening. The characteristics of the confirmed cases are presented in Table 2. The overall 30-day all-cause mortality rate was 1.65% (95% CI, 1.49–1.82). The confirmed patient cases detected by universal admission screening had a significantly lower proportion of 30-day all-cause mortality (2.18% vs 3.59%; *P* < .01), younger age (54 years vs 59 years; *P* < .01), and a higher proportion of fully vaccinated patients (72.4% vs 62.7%, *P* < .01), than confirmed patient cases detected by symptom-based testing. Companions were significantly younger, had a higher proportion of females, numbers receiving full vaccination, and a lower proportion of 30-day all-cause mortality patients (all *P* < .01) than patient cases, in both cohorts detected by universal admission screening and symptom-based testing.

**Table 2.** Characteristics of COVID-19 Cases Identified by Universal Admission Screening and Follow-Up Symptom-Based Testing for Patients and Companions in Hospitals in Taiwan, May 2021–June 2022

Characteristic	Positive Test Results		Total	*P* Value
	Universal Admission Screening	Follow-Up Symptom-Based Testing^a^		
**No. of new cases**				
Patients	14,640	1,281	15,921	…
Companions	5,372	908	6,280	…
**Age, y**				
Patients, median (IQR)	54 (36–68)	59 (43–70)	55 (37–68)	<.001
Companions, median (IQR)	50 (38–62)	47 (38–59)	50 (38–61)	<.001
**Sex, female**				
Patients, no. (%)	7,247 (49.5)	597 (46.6)	7,844 (49.3)	.047
Companions, no. (%)	3,620 (67.4)	617 (68.0)	4,237 (67.5)	.737
**30-d all-cause mortality**^**b**^				
Patients, no. (%)	319 (2.2)	46 (3.6)	365 (2.3)	.001
Companions, no. (%)	1 (0.0)	1 (0.1)	2 (0.0)	.153
**Fully vaccinated against COVID-19**^**c**^				
Patients, no. (%)	10,594 (72.4)	803 (62.7)	11,397 (71.6)	<.001
Companions, no. (%)	5047 (94.0)	842 (92.7)	5,889 (93.8)	.160

Note. COVID-19, coronavirus disease 2019; IQR, interquartile range; RT-PCR, reverse-transcription polymerase chain reaction.

a Follow-up symptom-based testing was defined as reverse-transcription polymerase chain reaction tests performed in individuals with suspected symptoms of COVID-19 within 7 days of negative results of universal admission screening.

b The 30-day all-cause mortality was defined as death occurring within 30 days after the date of the first positive RT-PCR test result.

c A person was defined as fully vaccinated 14 days after a patient received a full primary series of COVID-19 vaccines.

**Figure 1.**
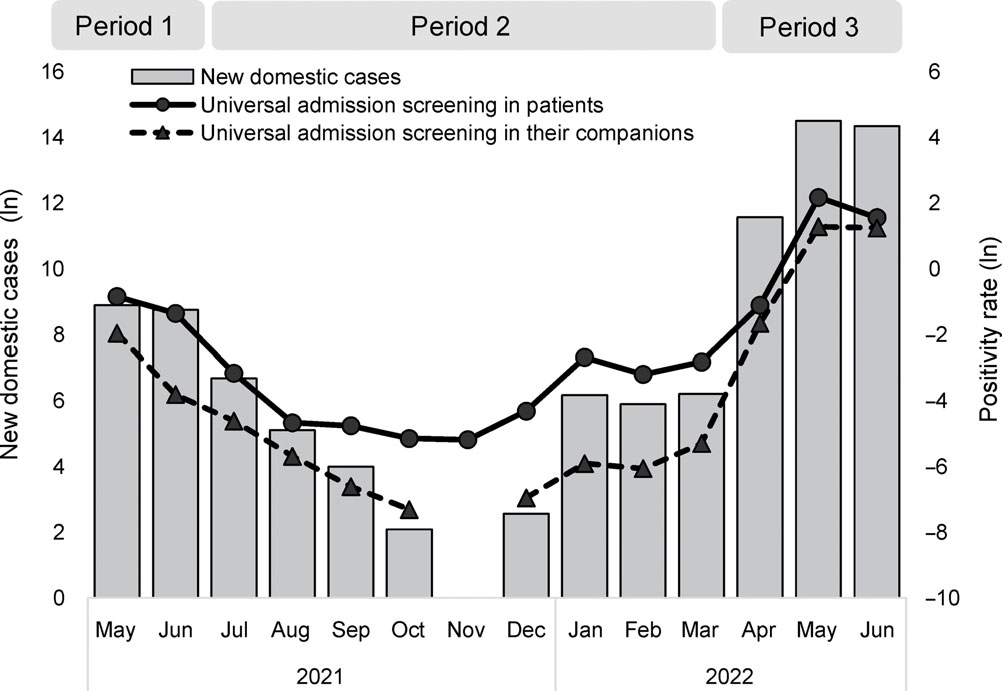
Incidence of coronavirus disease 2019 and monthly positivity rate of universal admission screening for SARS-CoV-2 in Taiwan, May 2021–June 2022. Bars show monthly new domestic coronavirus disease 2019 cases after logarithm transformation. The solid and dotted lines show the positivity rates of universal admission screening for SARS-CoV-2 after logarithm transformation in patients and their companions, respectively.

**Figure 2.**
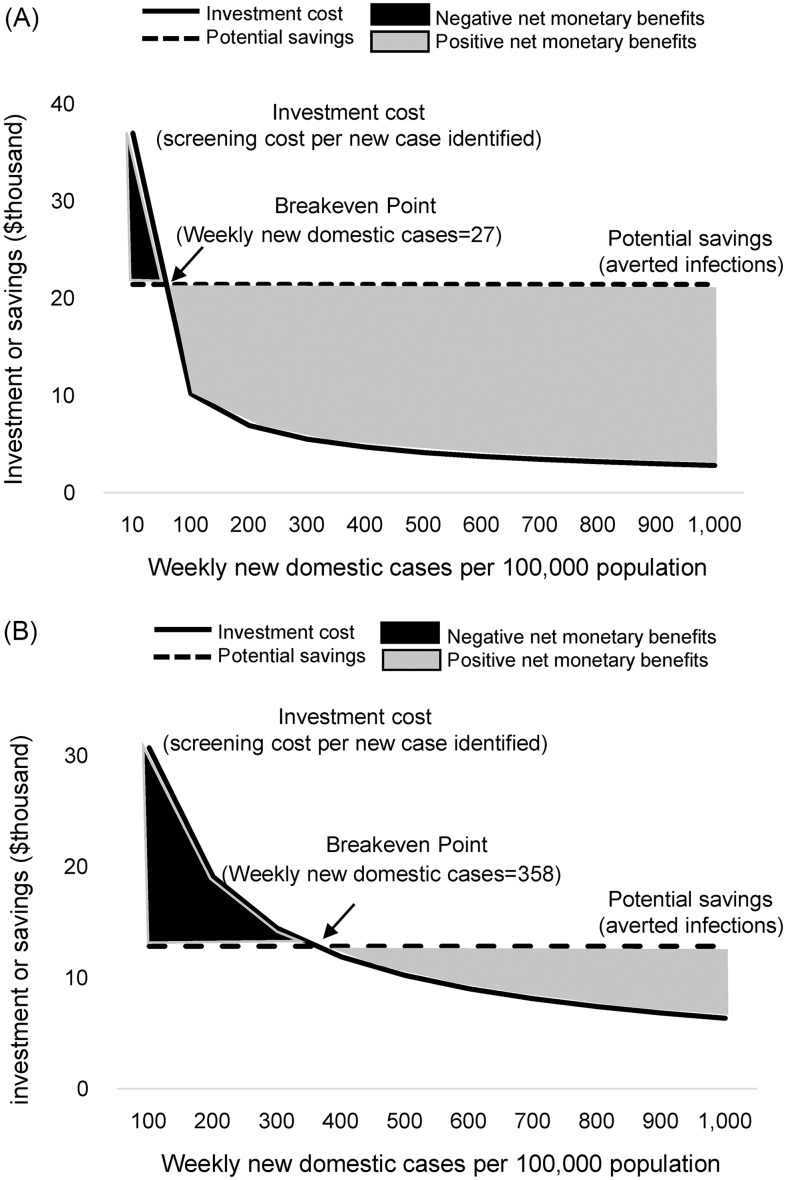
The investment costs and potential savings of universal admission screening for SARS-CoV-2 by community incidence of coronavirus disease 2019: (A) patients and (B) companions. The investment costs or potential savings of universal admission screening in thousands of US$ are depicted on the vertical axis, and the weekly community incidence rates of coronavirus disease 2019 (COVID-19) are shown on the horizontal axis. The solid line represents the investment costs of universal admission screening, calculated by the numbers needed to test multiplied by testing costs. The dotted line represents potential savings provided by universal admission screening, calculated by the medical cost of averted infections plus the testing cost of averted close contact. The breakeven point of universal admission screening was defined as a screening investment equal to potential savings. The black and gray zones represent the negative and positive net monetary benefits of universal admission screening, respectively.

The positivity rates of universal admission screening for patients and companions were positively correlated with the weekly community incidence rate of COVID-19 cases, with a correlation coefficients of 0.93 and 0.97, respectively (*P* < .01) (Fig. 1). The potential cost savings of an averted healthcare-associated COVID-19 outbreak initiated by a patient case and a companion case amounted to $21,413 and $12,811, respectively; these were approximately equal to the costs of universal admission screening at NNTs of 212 and 128. Thus, implementing universal admission screening for patients and companions when the weekly community incidence rate was 27 per 100,000 population with a positivity rate of 0.47% and 358 per 100,000 population with a positivity rate of 0.78%, respectively, would have achieved the breakeven point (Fig. 2).

## Discussion

Universal admission screening for COVID-19 has been adopted as infection control management to prevent in-hospital transmission of SARS-CoV-2.^3,4,9,11,13,16^ To the best of our knowledge, this was the first nationwide population-based cohort study to investigate the performance of universal PCR-based screening for SARS-CoV-2 in newly admitted patients and their companions during periods with varied community incidence rates.

The positivity rate of universal admission screening in newly admitted patients was 0.76% during a period with an average weekly community incidence of 270 per 100,000 population. Previous studies reported that the positivity rate of universal admission screening for SARS-CoV-2 ranged from 0.005% to 13.7%.^4,9,10,16^ The wide range of positivity rates in different studies was likely due to the different definitions of universal admission screening and community incidence. The positivity of universal admission screening for SARS-CoV-2 has been positively associated with the contemporary community incidence.^3,5^ We also observed a similar correlation; the positivity rate of universal admission screening in period 3 with greater community transmission was significantly higher than the positivity rates in periods 1 and 2.

The advantage of universal admission screening is that it provides information for hospitals to implement appropriate IPC measures in advance.^11,13,16^ Nevertheless, concerns about the potential drawbacks of universal admission screening remain, such as unnecessary isolation of individuals with false-positive test results and delay of necessary treatment for cases.^7,11,14–17^ Therefore, in the era of vaccination, it is debatable whether universal admission screening is still necessary, especially in areas with a low community prevalence of SARS-CoV-2.^5,7,10,11,17,22^ All positivity rates of the periods observed during this study were less than the prevalence threshold of 10% recommended by international academic associations for the implementation of universal testing for asymptomatic hospitalized patients.^7,11^

Although several studies supported universal admission screening, none of these were based on the intervention with cost and effectiveness assessments.^3,4,13,14^ Krüger et al^3^ reported that the NNTs and average costs of universal admission screening to detect an asymptomatic SARS-CoV-2 patient in a Germany tertiary-care hospital were 425 patients and ∼€25,075 ($28,247) in the high-incidence phase, with a positivity rate of 1.41% and 1,218 patients and ∼€71,862 ($78,969) in the low-incidence phase, with a positivity rate of 0.25%. Nevertheless, no exact beneficiary benefits of the screening strategy were observed in this study. Many studies have examined the cost-effectiveness of various test strategies for SARS-CoV-2, but studies focusing on universal admission screening are limited.^23–25^ Stevenson et al^24^ modeled 30 SARS-CoV-2 test strategies for patients admitted from the emergency department, with a community prevalence of 5.3%. These researchers found that the least costly test strategy was testing on hospital admission with retesting 6 days after admission, and they recommended that using tests with shorter turnaround times would be more cost-effective.^24^ Another modeling study in a simulation with a prevalence of 15.6% in Germany showed that testing patients in emergency departments before hospitalization reduced the average cost of hospitalized patients by €213 ($234) per tested patient.^25^ Instead of conducting a modeling cost–benefit analysis, we used real-world operational data to approximate costs and potential savings from averted healthcare-associated SARS-CoV-2 infections. Our results demonstrated that implementing universal admission screening for patients and their companions would achieve monetary benefits in the phase with weekly positivity rates of 0.47% and 0.78%, respectively. Thus, the costs of universal admission screening would be outweighed by the benefits only in period 3 of our study from a monetary perspective.

Cost-effectiveness is one of the dimensions of the decision-making process when evaluating interventions. Factors such as dominant virus variants in circulation, lack of appropriate vaccinations and pharmaceuticals, and the surge in the number of patients requiring care from the healthcare system should also be considered to justify universal admission screening during the early period of the COVID-19 pandemic.^3,6,16,17,23^ As the pandemic evolved, the virulence of the variants changed, effective vaccinations and antiviral agents were developed, and the immunity of the population increased.^16,21,26,27^ Therefore, the extra benefits of universal admission screening added to the existing hierarchy of IPC measures (eg, the appropriate use of PPEs, active health surveillance of healthcare workers, optimal unit layouts with enhanced ventilation) might need to be re-evaluated.^16,28–30^ The Society for Healthcare Epidemiology of America and the American Society of Anesthesiologists did not recommend routine universal testing for SARS-CoV-2 in asymptomatic patients at the time of hospital admission and before hospital procedures.^28,29^ Healthcare facilities should implement universal admission screening based on individualized risk and benefit assessments and should consider metrics such as the population at risk for severe infection (eg, residents of long-term care facilities, immunocompromised individuals), vaccination status, physical layout of the unit, and community transmission of SARS-CoV-2.^16,29,31,32^

Despite the infectivity of asymptomatic COVID-19 cases, interviews with all patients regarding symptoms through clinical triage and targeted testing would still be an important measure to detect SARS-CoV-2 infections.^3,4,11,16^ Follow-up symptom-based testing detected ∼9.86% of the confirmed cases in our study within 7 days after negative universal admission screening test results. These patients might have been in the incubation period when they received the admission screening, or they were infected during hospitalization, considering that the mean incubation period of SARS-CoV-2 is ∼5 days.^6,11,16^ This factor highlights the importance of clinical judgment for inpatients who develop symptoms compatible with COVID-19 during hospitalization. IPC teams in hospitals who implement universal admission screening should be aware of the false sense of security provided by the screening strategy and remind firstline healthcare workers of the significance of clinical assessment.

In our study, patients identified by symptom-based testing had a higher proportion of 30-day mortality than patient cases identified by universal admission screening. Other studies noted similar findings, but no specific explanations were provided in the discussions.^30,31^ Although we did not evaluate the underlying medical conditions of the cases, older age and a lower proportion of fully vaccinated individuals in patient cases identified by symptom-based testing may partially explain the higher mortality.^1,22^ A certain proportion of cases detected by universal admission screening might only be identified due to prolonged virus shedding, representing an unknown past infection instead of an acute infection. The majority of blood tests in asymptomatic cases in Malundo’s study were within normal limits.^30^ Some asymptomatic cases identified by universal admission screening possibly were caused by persistent shedding of nucleic acid fragments from past infections and this might also have contributed to the lower mortality of the group.

This study had several limitations. First, our data were retrieved from the NHI claims database, and the billing codes used for the tests were not validated. There was a possibility of patient or provider bias when stating or collecting data about the presence or absence of symptoms, thus potentially influencing the type of billing code selected. Second, we did not conduct a formal cost–benefit analysis, and many parameters that should have been included in such modeling studies were not considered, such as the protective effectiveness of vaccination, performance of the test kits, indirect costs incurred by ward closure, and productivity loss incurred by quarantine or isolation. Consequently, potential savings might be underestimated or overestimated; therefore, further studies are warranted. Nevertheless, we used observation data from the operations of Taiwan healthcare systems in the real world, which may still provide useful information for the decision-making process of relevant policies in the future. Third, we did not have the RT-PCR cycle-threshold value results, which is a commonly used proxy for COVID-19 infectiousness. Therefore, we were not able to discriminate between cases identified by universal admission screening as acute or past infections, which could have resulted in the overestimation of the benefits of universal admission screening. Finally, because of the well-operated national laboratory networks for SARS-CoV-2 testing in Taiwan, each hospital could perform universal admission screening with RT-PCR either alone or in collaboration with designated laboratories. Therefore, our results might not be generalizable to countries or regions with different diagnostic capacities.

Controlling SARS-CoV-2 transmission in hospitals is critical for reducing deaths and severe illnesses from COVID-19. Universal admission screening of patients and their companions can control the spread of asymptomatic and presymptomatic SARS-CoV-2 infection in healthcare settings. By determining the appropriate timing and targets for the implementation of this strategy, the benefits can be balanced with the potential unintended harms, and resources can be allocated more efficiently.
